# Mind over matter. The impact of subjective social status on health outcomes and health behaviors

**DOI:** 10.1371/journal.pone.0202489

**Published:** 2018-09-05

**Authors:** Lorenzo D’Hooge, Peter Achterberg, Tim Reeskens

**Affiliations:** Department of Sociology, Tilburg University, School of Social and Behavioural Sciences, Tilburg, The Netherlands; University of Michigan, UNITED STATES

## Abstract

Recent insights have shown subjective status to impact health and health behavior. It is however unclear how this exactly happens. In this study we explore two mechanisms: this of a direct, mediating effect of subjective status explaining the impact of material class on health outcomes and behavior and an indirect, moderating impact on the relationship between material class and health outcomes and behavior. To test this empirically we conduct two studies, focusing on Great-Britain, using survey-data from the English Longitudinal Study of Ageing (N: 2709–3448) and the Whitehall II-study (N: 6275–6467). Our linear and logistic regression analyses show subjective status has a mainly direct impact on health outcomes and has both a direct, mediating and indirect, moderating impact on health behavior. In the conclusion of our article we reflect on the theoretical reasons why subjective status has a direct impact in certain cases, while playing an indirect role in other cases.

## Introduction

For several decades, studies found a positive relationship between socioeconomic positions and health [1], [2], [3], [4], [5], [6] and what the literature refers to as health lifestyles [7], [8], [9] described as “collective patterns of health-related behavior based on choices from options available to people according to their life chances” [10]. Theoretically, this link should be strong and robust because social positions are grounded in the relationship between the individual and the market; as such, social classes combine people with shared market positions and, concomitantly, similar life chances [11], [12], [13]. Partly due to their less favorable life chances, those lower in the hierarchy tend to lead unhealthier lives than their counterparts higher up [14], [15]. This social gradient is a cumulative process over the life course and is not limited to only the poorest; also the middle class is more affected by poorer health than those at the top of the socioeconomic ladder [16], [17], [18], [19].

Although research on these material class positions has delivered seminal insights into inequalities in health [19], [20] and health behaviors [7], sociologists increasingly point at the relevance of subjective class identification as a relevant predictor [21], [22] While this idea in itself is promising, no previous studies have focused on disclosing *how* subjective perceptions of one’s social position exactly play a role. Earlier work [21] suggests that a priority for further research should be to clarify the causal mechanisms explaining how a cultural interpretation of social position influences the relationship between material class and health/behavior. In this paper we aim to fill in this black box in two ways. First of all, we focus on *how* subjective perceptions exactly play a role, precisely because the pathways through which material and subjective positions influence health outcomes have not been adequately explored. Secondly, we aim to extend research on the importance of subjective social status further by also focusing on health behaviors since earlier studies [21], [22] mostly tapped into the possible relevance of subjective perceptions in regards to health itself.

We propose two distinct mechanisms through which subjective status plays a role. Firstly, subjective status can have a *direct*, *mediating* effect on health and behavior as a consequence of an individual’s material position [22], [23]. Several studies argue that the structural or material aspect of class influences the cultural part, meaning that subjective perceptions are a consequence of material positions [24], [25]. Since people often do not perceive their position as it materially is [26], [27], subjective status can be a predictor of health and health behavior since it mediates the role of material class. While this mediation has received some attention in earlier research on health [21], [22], we also propose a second mechanism through which subjective status can play a role. Inspired by the idea put forward by [28] entailing that only when individuals *believe* they belong to a material class this class can be of importance, we expect one’s material class position only to be influential when that person perceives it in a similar way. This means that subjective status can *indirectly* affect health and behavior by *moderating* the relation between one’s material class and health/behaviors.

This is fundamentally different from the first mechanism since it focuses on *when* material class and subjective perception are important rather than solely on whether subjective status explains (part of) the effect of material class. In this moderating pathway, material class plays a role when people perceive their position as such while it loses its importance when this is not the case. Where the first mechanism expects subjective status to explain (part of) the importance of material class, the second mechanism anticipates a situation in which the health or behavior outcomes differ *within* a material class according to one’s perceptions of their status.

To empirically test these two mechanisms we conduct two distinct studies. We prefer this set-up over one methods and results section in order to make our findings more comprehensible. Since we use two distinct data-sources, because no overarching dataset answering all of our questions is available, describing the specifics, used methods and found results separately instead of in one section, grants the reader a clearer view on the specific variables and results in regards to health outcomes and health behaviors. In study 1, we focus on a number of biomarkers, where higher levels indicate a higher risk on developing cardiovascular diseases (systolic and diastolic blood pressure, cholesterol, triglycerides, high-sensitivity C—reactive protein and hdl-cholesterol) from the English Longitudinal Study of Aging (ELSA) which includes information of 3,559 British people over 52 on a number of health indicators in addition to material and subjective social position. Since the ELSA-data does not contain adequate information on health-related lifestyle we use data from the Whitehall II survey in the second study, which contains information on 6,501 civil servants regarding their material and subjective social position in addition to health behavior (alcohol consumption, exercise, smoking, and food choices). While the ideal empirical set-up would include information from representative samples for the whole population, by focusing on two distinct subsections–the elderly and civil servants from London–we cannot give a complete view; nevertheless, in the absence of such ideal sources both our used datasets are unique because of their inclusion of material class, subjective status and detailed information on health biomarkers/behaviors.

## The impact of material class and subjective status on health and health behavior

While genetic and personal circumstances are important when it comes to predispositions in health outcomes [29], social science research untangled that two aspects of social position play a vital role, too, namely material class and subjective status. The material conditions in which people live their lives impact health outcomes [16], [17], [29], [30] causing those lower in the social hierarchy to have more health problems, while those higher up demonstrate the opposite [29], [31], [32]. This difference is not limited to those with a lack of means to buy appropriate medicine or undergo treatment but affects all layers of society [17]. Not only are the poor in a more precarious condition than the rich; also those in the middle differ from those lower or higher up in society [16], [18], [33]. Furthermore, the social gradient in health not only appears in biomarkers, but is also present in health behaviors. Studies show a pattern of unhealthier lives with more alcohol abuse, more smoking, less exercise and unhealthier food in the lower material classes [34], [35]. Whereas healthy or unhealthy life choices are noticeable at any given age, the social gradient regarding health is more discernible at later age [19]. While childhood influences future health [36], [37], circumstances later in life during adulthood have a fundamental impact [32], [38]. Health is thus a result of an aggregate process of contextual influences people undergo in their lives [39]. While the social gradient in health outcomes has been a robust finding in previous research [2], [40], [41], [42]; recent studies indicate that the causal mechanism goes beyond monetary explanations and should be found in cultural reasons as well. The scholarly attention for subjective social positions has been somewhat lacking [43] making its role in the social gradient in health and health lifestyle to be not fully entangled. As already established decades ago [23], [44], it is not only the material position one occupies but also the personal perception of it that can have a profound effect on one’s life (see also [45], [46], [47]). Indeed, these subjective perceptions relate to health [21],[22] and health lifestyle [48], [49], [50], yet the pathways through which this happens are not fully clear.

Social identity theory [51] explains that an individuals’ social identity is “that part of an individual’s self-concept which derives from his knowledge of his membership of a social group (or groups) together with the emotional significance attached to that membership” [51]. Additionally, social categorization theory [52] suggests a de-personalization of the self [53], implying that people who see themselves as part of a certain group share similar behavior [7], [54], including health behavior and according health outcomes [21], [22]. Since people try to adhere to favorable feelings towards the own group [52], [55], lower perceptions of one’s social status results in health outcomes and behavior in accordance with individuals with a similar subjective status irrespective of one’s same material class [22], [56]. Furthermore, we can also draw on the Thomas theorem: “if men define situations as real, they are real in their consequences” [57] in [58]. People who perceive their social position in a certain way, irrespective from the class they materially belong to, will have different health outcomes and behavior than others in their material class with a different perception. The perception people have concerning their social status does not only influence explicit aspects as their behavior, such as their health behavior, because they follow what they expect is the norm of the group they feel they belong too but also extends a more subtle influence on the way they handle information, seek treatment and follow advice from medical professionals resulting in better or worse health [39], [59].

However, how people experience group membership does not always coincide with how it materially is. Empirical studies have shown subjective perceptions to often differ from material class [26–27]. While the reasons why subjective identification does not collapse with material class position are relevant to study (see [26] assessing causes ranging from status inconsistency to cross-cutting social identities), this is beyond the scope of our inquiry. In regards to research on health, however, some studies indeed do suggest subjective perceptions to be important regarding disparities in health [5], [6], [32] but it is unclear in which way this occurs and whether this extends to health behaviors. The fact that subjective social status could impact health both through a mediating and possibly moderating pathway [21] is the basis of our empirical scrutiny. Additionally, we extend our study towards health where experimental studies indicate subjective social status to be of importance [48], [49], [50].

We propose two distinct mechanisms offering an explanation; first, there are scholars describing one’s material position to play a definite role in how people see their social position in society, i.e. their subjective status [5], [60], [61], [62]. Since people form an idea of their place in the social stratification by virtue of small networks, meetings with co-workers, unions or employer organizations, and connections with other people in similar positions [63], [64], by means of mediation subjective status is expected to act as a predictor of health and behavior next to material class since part of the population perceives their position as different from what it is [26–27]. Secondly, a cultural sociological approach states that subjective status moderates the influence of material class on health outcomes and behaviors since material social position can *only* be of importance if one subjectively believes that one *is* in such a social position [28]. According to this logic the subjective status individuals have is not necessarily influenced by their material class but rather impacts the relationship between material class and health/behavior since people adapt to what they believe are the expectations of the social position they perceive themselves to be part of [7], [54]. Where the first mechanism anticipates subjective social status to explain (part of) the role of material class, the second mechanisms anticipates how subjective social status impacts the relationship between material social class and health outcomes/behaviors.

**Fig 1:**
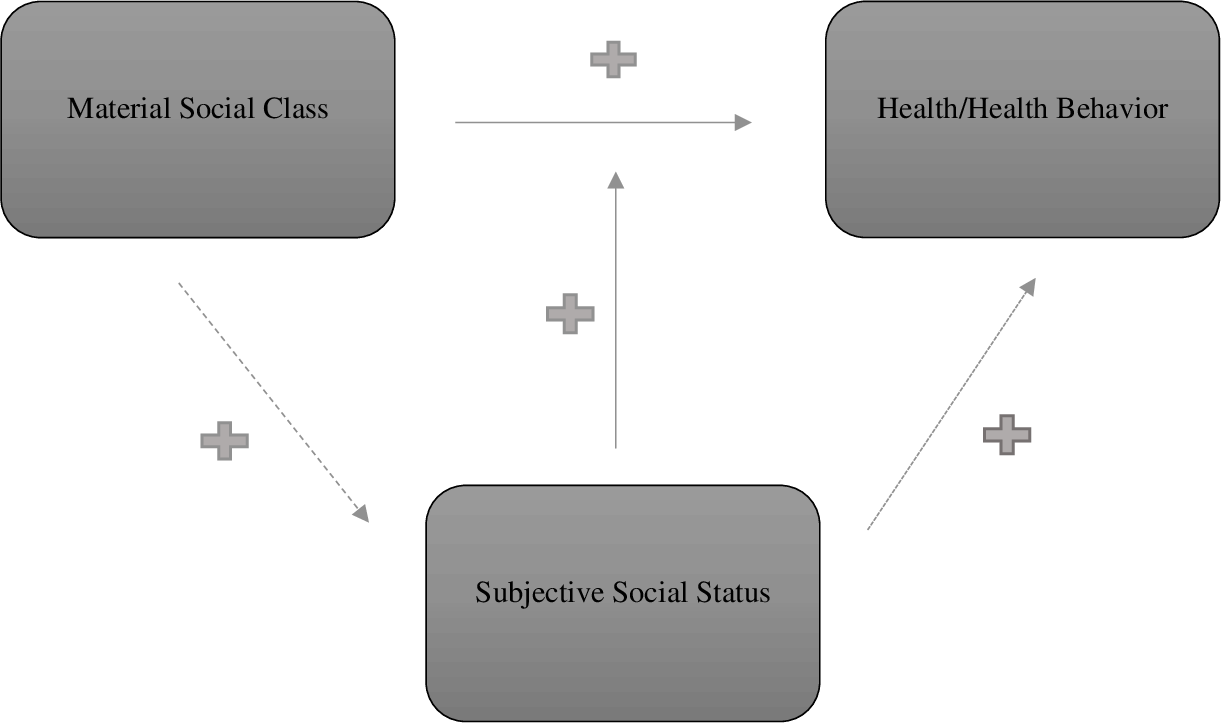
Conceptual model with the hypotheses regarding mediation and moderation.

Following the Thomas theorem and the literature [14], [17], [32], [65], we formulate two distinct sets of hypotheses (see Fig 1). First, concerning *health biomarkers* we expect those with a lower material social class to be unhealthier. We do however expect subjective social status to be important through two pathways. On the one hand we expect subjective status to mediate the effect of material class, with a higher subjective status resulting in better health outcomes and a lower subjective status being associated with worse health outcomes. On the other hand, we anticipate subjective social status to moderate the role of material social class resulting in better health outcomes within a material class when one’s subjective status is higher, and worse health outcomes within a material class when one’s subjective status is lower.

Regarding *health behavior* we expect those with a lower material class to display unhealthier behavior, but when accounting for subjective social status we expect the latter to mediate the impact of the former, expressing itself as those having a higher subjective status acting healthier and those with a lower subjective status doing the opposite. Furthermore, we again have an alternative cultural sociological hypothesis where we anticipate the effect of material social class on health behavior to be influenced by subjective social status. While those with a lower material class will display more unhealthy behavior, this can be counteracted by a higher subjective social status. On the other hand, those with a higher material class are expected to have less healthy behavior when their subjective status is lower.

## Study 1

### Data & methods

#### a. Sample

In our first study, data from the second (2004/2005) and sixth (2012/2013) wave of English Longitudinal Study of Ageing is used to assess health outcomes by means of blood pressure and several biomarkers (cholesterol, triglycerides, high-sensitivity c-reactive protein and high density lipoprotein-cholesterol) in the blood of the respondents. Information on material class, measured through occupation, and subjective status from the second wave (2004/2005) is used to predict health outcomes, 8 years later, measured in wave six (2012/2013). We use a time lag because of the potentially delayed impact of material and subjective position on health, although we also do a robustness check using only data from wave six. The correlation between material class in wave 2 and wave 6 is 0.90, while the correlation between subjective social status in these two waves is 0.60, illustrating some change for the latter. The distribution/average and standard deviation for both, however, is virtually unchanged meaning that only few people have changed their material class position while for subjective social status some people have increased their subjective status while for others it has decreased. The data was collected by face-to-face interview and nurse visits. In our first table estimating the effect of material class and subjective social status on our health index, we use 3,588 cases. While the number of cases is the same within each set of Models, they can differ upon the specific dependent variable. The exact number of cases is mentioned in the tables for each dependent variable.

#### b. Independent variables

In the ELSA-sample, *material class* is measured by a five-category scheme measuring occupation, resembling the EGP-scheme that is based on the occupational activity, its level of authority and type of labor-contract [66]. The survey distinguishes between (1) Managerial and professional occupations, (2) Intermediate occupations, (3) Small employers and own account workers, (4) Lower supervisory and technical occupations and (5) Semi-routine occupations. Based on earlier work [67] showing that aggregating managerial and professional occupations as higher class and the (semi-)routine occupations as the lower class does not lower the validity, we aggregate these five classes to three. We opt to do this because it makes the interpretation of the results easier while not resulting in a loss of information. As indicated in S1 Table, we reduce these five categories to three material classes: (1) the higher class (managerial and professional occupations), (2) the middle class (comprising of intermediate occupations, small employers and own account workers and lower supervisory and technical occupations) and (3) the lower class or working class (semi-routine occupations). Those who had never worked, were long time unemployed and housewives are excluded from the analyses. About 37.5% of the sample is higher class, while respectively about 34.5% is middle class (13.5% intermediate occupations, 11.2% small employers and own account workers and 9.9% lower supervisory and technical occupations) and about 28% working class.

*Subjective social status* is measured by the MacArthur scale of subjective social status [68], [69] asking individuals where they place themselves on a ladder ranging from 0–10 with those worst off in society at the bottom and those best off at the top. In our study we reverse this scale so that a higher score implies a lower subjective status and a lower score implies a higher status. We do so in order to have our indicators of material class and subjective class both coded in the same direction, making interpretation easier. We test the role of subjective social status both in a linear way as in a categorical way where we trichotomize the MacArthur scale in three groups each containing approximately one third of the sample. This allows us in first place to assess whether subjective social status has a linear effect or not and secondly when estimating a potentially moderating role of subjective social status this allows us to match these groups with the material social classes. This helps to assess whether subjective social status has a different effect for those whose subjective social status is concordant versus non-concordant. The descriptives of all variables are presented in S1 Table.

#### c. Dependent variables

Health is measured by the following biomarkers: measured systolic and diastolic blood pressure, total cholesterol, high-density-lipoproteïn cholesterol, high sensitivity C-reactive protein and triglycerides. These biomarkers are found in blood samples obtained by a visiting nurse and are dichotomized as being healthy (0) or unhealthy (1). In doing so we follow earlier studies using these biomarkers who dichotomize these variables since a linear effect is hard to interpret in terms of being healthy or unhealthy. As a robustness check we do however estimate our models with linear variables. The cut-off points derived from earlier studies [22], [70], are >140mmHG for systolic blood pressure, >90mmHG for diastolic blood pressure, >6.2mmol/l for cholesterol, <1.03mmol/l for HDL-cholesterol, >3mg/l for hs-CRP and >2.26 mmol/l for triglycerides. When respondents take medication to control their blood pressure they are categorized as unhealthy on the systolic and diastolic blood pressure-biomarker, while they are categorized as unhealthy on the total cholesterol-biomarker when they take cholesterol medication. In both cases these respondents are coded as unhealthy regardless of their measured blood pressure/level since when it is in the healthy range it is a consequence of their medication.

In a first step, we construct an index of health where a higher score implies a poorer health and a lower score acts as an indication of a better health. Here the score a respondents gets ranges between 0 and 6, where the former refers to respondents with no biomarkers above the respective unhealthy thresholds and the latter refers to individuals with all biomarkers above the respective unhealthy thresholds. In a second step in S2 Table, we analyze the biomarkers separately to provide a more nuanced view on how material class position and subjective social status are related to these health outcomes.

#### d. Control variables

We control the analyses for *level of education* since higher educated individuals tend to be healthier [40], [64]. We distinguish between (0) people without a post-secondary education or (1) people who do have a post-secondary education. Furthermore, we control for *gender*, *marital status* and *age*. Since *cholesterol medication* can also unintentionally lower the levels of triglycerides, hs-CRP and HDL, we control for this [71], [72], [73]. We do not respectively include these control variables in the models on total cholesterol and blood pressure since those taking such medication are already coded as having unhealthy levels in the separate models in S2 Table.

#### e. Method

First, when testing our index of health in Table 1 we use multivariate linear regression models. Second, in S2 Table we estimate models for the separate health biomarkers, where we use binomial logistic regression models to measure how material class and subjective status impact them, since these are dichotomized to healthy or unhealthy. When doing so we look at both a direct, mediating and an indirect, moderating effect of subjective status. To test this mediating effect we use the Karlson-Holm-Breen Method [74] as a significance test for the potentially confounding effect of subjective social status on the relationship between material class and health outcomes. The KHB-package [75] for Stata allows to express the occurrence of mediation as the % of the variation that is explained by the mediator and whether this is significant. In our case, this allows to test whether subjective social status mediates the effect material class has on health in general and the separate health biomarkers.

**Table 1:** Material class and subjective social status regressed on health biomarkers with control variables.

N: 3588	Model 1	Model 2	Model 3	Model 4	Model 5
**Constant**	1.063 (0.17)***	1.248 (0.20)***	0.937 (0.18)***	1.383 (0.23)***	0.932 (0.18)***
**Material Class**					
Higher Class (Ref.)	-	-	-	-	-
Middle Class	0.028 (0.05)	0.000 (0.05)	0.003 (0.05)	-0.300 (0.20)	
Working Class	0.028 (0.05)	-0.000 (0.05)	0.008 (0.05)	-0.067 (0.19)	
**Gender**					
Male (Ref.)	-	-	-	-	-
Female	-0.047 (0.04)	-0.025 (0.04)	-0.026 (0.04)	-0.029 (0.04)	-0.032 (0.04)
**Education**					
Lower education (Ref.)	-	-	-	-	-
Post-secondary education	-0.182 (0.05)**	-0.128 (0.06)*	-0.140 (0.06)*	-0.124 (0.06)*	-0.137 (0.06)*
**Marital Status**					
Married (Ref.)	-	-	-	-	-
Not-Married	0.040 (0.04)	0.037 (0.04)	0.043 (0.04)	0.037 (0.04)	0.042 (0.04)
**Age**		0.006 (0.00)	0.006 (0.00)*	0.006 (0.00)*	0.006 (0.00)*
**Decreasing Subjective Status**		0.004 (0.00)***			
**Subjective Status Groups**					
Higher status (Ref.)	-	-	-	-	-
Middle status			0.036 (0.05)		
Working status			0.138 (0.05)**		
**Material Class X Subjective Status**					
Higher X Subj status (Ref.)	-	-	-	-	-
Middle X Subj Status				0.005 (0.00)	
Working X Subj Status				0.001 (0.00)	
**Class-Status Combinations**					
Higher class-Higher status (Ref.)	-	-	-	-	-
Higher class-Middle status					0.029 (0.08)
Higher class-Lower status					0.258 (0.08)**
Middle class-Higher status					0.103 (0.07)
Middle class-Middle status					0.092 (0.08)
Middle class-Lower status					0.070 (0.07)
Working class-Higher status					-0.036 (0.09)
Working class-Middle status					0.061 (0.08)
Working class-Lower status					0.205 (0.07)**
**R^2^**	0.57%	0.95%	0.88%	1.02%	1.10%
**Karlson, Holm, Breen-Mediation Analysis**					
**% of Material social class mediated by Subjective social status**					
Higher Class		-	-		
Middle Class		97.93%**	88.89%*		
Working Class		100.64%***	79.27%**		

Source: Wave 2 and 6 of the English Longitudinal Study of Ageing.

° p > 0.10.

* p < 0.05.

** p < 0.01.

*** p < 0.001.

### Results

In our study on health measured by biomarkers indicating cardiovascular health, we formulated two hypotheses on alternative mechanisms explaining the role of subjective status. On the one hand, we expected a mediating relationship with health outcomes, explaining the effect of material class. Moreover we anticipated a moderating effect where subjective social status alters the effect of material class.

The results on our index of health in Table 1 remarkably show no direct association between material class position and health in Models 1–3. Model 2, however, shows this to be the case for subjective social status, since a lower status is associated with poorer health. Model 3, further illustrates that there is mainly difference between those in the highest and lowest subjective status group, with the latter having a poorer health. Additionally, we also zoomed in on the relationship between material class and subjective status and found a weak correlation of 0.30, explaining why material class bears no importance while subjective social status does. Remarkably, we find a small moderating effect in Model 5, where we see that those assessing their status to be lower regardless of whether they are materially part of the working or higher class are equally unhealthier than those materially part of the higher class who assess their subjective status to be high. This small moderating effect also explains why there is a significant mediation effect of subjective social status in Models 2 and 3. While this may seem puzzling since there is no direct effect of material social class, these findings illustrate that material social class can have an indirect effect on health through subjective social status. Using a powerful mediation analysis such as the KHB-method in contrast to the causal steps-approach [76] allows to reveal such mediation even when there is no direct effect [77].

The results in Models 1A-6A of S2 Table somewhat nuances the findings in Table 1 by showing that the social gradient does exist, but not for all biomarkers. While a direct effect of material social class is absent when studying an index of health in Table 1, we see in S2 Table that those in the working class are more likely to have an unhealthy systolic and diastolic blood pressure, next to having unhealthy levels of hs-CRP. No relationship is found with cholesterol, triglycerides and HDL-cholesterol. Furthermore, Models 1B and 4B show a significant mediating effect of subjective social status on the likelihood of having an unhealthy systolic blood pressure or an unhealthy level of triglycerides although in the latter case there is no significant main effect of material class. Additionally, Model 6B illustrates, while there is no impact of material class, a lower subjective status is associated with a higher likelihood of unhealthy levels of HDL-cholesterol. Neither material class nor subjective status is related to overall cholesterol-levels. Furthermore, we see that the earlier found small moderating effect of subjective social status does not emerge for any of the specific biomarkers.

To summarize, our results confirm a higher subjective social status to be associated with better health although our more nuanced models show this to depend on the exact biomarker. Remarkably, when assessing health we do not find a relationship with material social class, meaning that we do not find the social gradient to be confirmed. However, again, in our more nuanced analyses in S2 Table we see that the existence of the social gradient depends on the specific biomarker. Here we also see that subjective social status in certain cases mediates the role of material social class, while for some biomarkers there is a small moderating role as well.

To validate our results further we did three robustness checks, of which the results are obtainable from the authors. Firstly, we redid our analyses on the level of triglycerides, hs-CRP and HDL-cholesterol without controlling for taking cholesterol medication. Doing so did not substantially alter our estimates. Secondly, we analyzed each biomarker as a linear effect instead of a dichotomous variable. This also did not substantially alter the results. Thirdly, we used material and subjective position from the same wave (being wave 6) as when the biomarkers are measured. This yields the same results expect for a significant effect of subjective social status on hs-crp and a small moderating effect of subjective status for HDL-cholesterol. In the former case, a lower subjective status is related to a higher chance of unhealthier levels of hs-crp, while in the latter case people in the working class are more likely to have unhealthy levels of HDL-cholesterol when their subjective status is lower. It is however unclear whether those people have unhealthier hs-crp and HDL-levels because of their lower subjective status or whether those who are unhealthier have a lower subjective status.

## Study 2

### Data and methods

#### a. Sample

In the second study we use data from the fifth wave (1997–1999) of the Whitehall II-survey to analyze health behavior (alcohol consumption, exercise, food habits and smoking). This survey is a longitudinal cohort study of British civil servants that started in 1984 and consists of eleven waves. While this data-source only includes residents of London, it is to our knowledge one of the best data-sources regarding health behavior. The data were collected by face-to-face interviews. Whitehall II data are available to bona fide researchers for research purposes. Please refer to the Whitehall II data sharing policy at http://www.ucl.ac.uk/whitehallII/data-sharing. In our first table estimating the effect of material class and subjective social status on our index of health lifestyle, we use 3,588 cases. While the number of cases is the same within each set of Models, they can differ upon the specific dependent variable. The exact number of cases is mentioned in the tables for each dependent variable.

#### b. Independent variables

In our sample *material class* already consists of the Administrative class, the Professional/Executive class and the Clerical/Support class. About 45% of the sample belongs to the Administrative class, while respectively 44% and 11% make up the Professional/Executive class and the Clerical/Support class.

*Subjective status* is again measured by the MacArthur scale (see [68] [69]) where individuals place themselves on a ladder ranging from 0–10 with those worst off in society at the bottom and those best off at the top. Respondents could also place themselves halfway between two ladders. As in the first study we reverse the scale so that a higher score implies a lower subjective status and a lower score indicates a higher status. Further, again following our first study, we also trichotomize subjective social status allowing to cross these groups with the material classes. The descriptives of all variables can be found in S1 Table.

#### c. Dependent variables

To study *health behavior* we construct an index of several health behavior ranging from 0 to 5 [78]. A lower score implies a healthier lifestyle while a higher score indicates an unhealthier lifestyle. In this index, we assess *alcohol consumption* by looking at the units of alcohol, wine and beer consumed per week since higher alcohol consumption is seen as unhealthier [79]. We use a cut-off point of 14 units a week to label respondents as drinking too much alcohol since drinking more has been linked to higher chances of several health issues [80], [81]. Because exercising is healthy [82], e*xercise* is measured through the self-reported amount of hours per week spent on moderate and vigorous exercise. We recode moderate and vigorous exercise into one variable measuring whether people exercise too little (less than 3 hours of moderate exercise or less than 1 hour of vigorous exercise [78] or whether they exercise enough. Further, unhealthy food choices are measured by looking at whether respondents prefer *whole wheat* bread over unhealthier options and whether they daily consume *fruits and vegetables* [83]. Finally, because of the negative health implications [84] we assess the likelihood of *smoking*. Respondents who don’t smoke or who have smoked in the past but stopped are coded as non-smokers, while those who currently smoke are coded as smokers. As a robustness check, we redid our analyses while coding those who had smoked in the past as smokers.

#### d. Control variables

We also include some control variables, namely *level of education* since schooling is related to knowledge about healthy living [21]. We distinguish between those (0) without post-secondary education and (1) with post-secondary education. Furthermore, we control for *gender*, *marital status* and *age*.

#### e. Method

We use two kinds of analyses to assess the role of subjective status. We use linear regression models to analyze our models on health behaviors in general. Further, to study in a more nuanced way how material class and subjective social status are related to the separate indicators of health behavior we use binomial logistic regression models. To estimate whether subjective social status has a mediating effect on the relationship between material class and our index of health lifestyle and the separate health lifestyles, we again employ Karlson-Holm-Breen Method [74].

### Results

In our second study we had two hypotheses concerning health behavior. First, we expected people with a lower material class to display unhealthier behavior but when accounting for subjective social status we expected the latter to mediate the effect of material class. Second, we had a cultural sociological hypothesis expecting the effect of material social class on health behavior to be influenced by subjective social status, through a moderating pathway.

In the results in Table 2, studying health lifestyle in general, we see in Model 1 that, contrary to the results on the health biomarkers, a lower material social class is associated with an unhealthier lifestyle confirming our expectation. Further, when assessing the relationship between material class and subjective social status we find a moderately strong connection of 0.56 while Models 2 and 3 show that subjective social status significantly mediates the effect of material social class, expressed by an unhealthier lifestyle for people who perceive their subjective status to be lower. Model 3 further illustrates that this is mainly a difference between those with a high status living healthier compared to those with a lower status living an unhealthier life, while those in the middle do not differ. Models 4 and 5 further illustrate a moderating effect of subjective social status since Professionals and Clericals live an unhealthier lifestyle when their status is lower and consequently live a healthier lifestyle when their status is higher.

**Table 2:** Material class and subjective social status regressed on unhealthy behaviors including control variables.

N: 6275	Model 1	Model 2	Model 3	Model 4	Model 5
**Constant**	3.191 (0.12)***	3.034 (0.12)***	3.190 (0.12)***	3.279 (0.16)***	3.196 (0.12)***
**Material Class**					
Higher Class (Ref.)	-	-	-	-	-
Middle Class	0.111 (0.03)***	0.044 (0.03)	0.052 (0.03)	-0.226 (0.09)*	
Working Class	0.257 (0.04)***	0.154 (0.05)**	0.166 (0.05)**	-0.120 (0.17)	
**Gender**					
Male (Ref.)	-	-	-	-	-
Female	-0.373 (0.03)***	-0.366 (0.03)***	-0.370 (0.03)***	-0.366 (0.03)***	-0.371 (0.03)***
**Education**					
Lower education (Ref.)	-	-	-	-	-
Post-secondary education	-0.122 (0.03)***	-0.099 (0.03)***	-0.108 (0.03)***	-0.110 (0.03)***	-0.108 (0.03)***
**Marital Status**					
Married (Ref.)	-	-	-	-	-
Not-married	0.120 (0.03)***	0.100 (0.03)**	0.104 (0.03)**	0.097 (0.03)**	0.102 (0.03)**
**Age**	-0.018 (0.00)***	-0.019 (0.00)***	-0.019 (0.00)***	-0.019 (0.00)***	-0.019 (0.00)***
**Decreasing Subjective Status**		0.048 (0.01)***			
**Subjective Status Groups**					
Higher status (Ref.)	-	-	-	-	-
Middle status			0.046 (0.03)		
Lower status			0.168 (0.04)***		
**Material Class X Subjective Status**					
Administrative X Subj status (Ref.)	-	-	-	-	-
Professional/Executive X Subj Status				0.064 (0.02)**	
Clerical/Support X Subj Status				0.061 (0.03)*	
**Class-Status Combinations**					
Administrative-Higher status (Ref.)	-	-	-	-	-
Administrative-Middle status					0.059 (0.04)
Administrative-Lower status					0.048 (0.07)
Professional-Higher status					0.019 (0.05)
Professional-Middle status					0.085 (0.04)*
Professional-Lower status					0.235 (0.04)***
Clerical-Higher status					0.304 (0.16)
Clerical-Middle status					0.155 (0.08)
Clerical-Lower status					0.336 (0.05)***
**R^2^**	4.14%	4.53%	4.48%	4.70%	4.57%
**Karlson, Holm, Breen-Mediation Analysis**					
**% of Material social class mediated by Subjective social status**					
Administrative Class		-	-		
Professional/Executive Class		58.55%**	52.00%**		
Clerical/Support Class		40.92%***	33.42%***		

Source: Wave 5 of the Whitehall II Study.

° p > 0.10.

* p < 0.05.

** p < 0.01.

*** p < 0.001.

Furthermore, we studied our indicators of health lifestyle separately as well and when observing the Models in S3 Table we can nuance the findings in Table 2. These models illustrate how the social gradient does not exist for every indicator of health lifestyle in the same direction. In some cases a lower material class position is associated with a higher likelihood of unhealthy behavior while in other cases the opposite happens.

In all Models a higher score on each dependent variable indicates unhealthier behavior. Models 1A-7A show differentiation between material classes. We see in Models 1A-4A that there is unhealthier behavior in the lower material classes regarding exercise, healthier food and smoking. When it comes to drinking too much alcohol however, Models 5A-6A demonstrate how those in a lower material class are less likely to consume too much alcohol in general or too much wine, while Model 7A shows those in the professional class are more likely to have an excessive beer consumption. The Administrative class in general lives a healthier lifestyle in regards to exercise and food choices, but is more likely to make unhealthy choices in regards to alcohol consumption. These findings nuances the findings on the social gradient in Table 2: whereas the social gradient clearly exists in regards to health behavior, it is in certain cases, related to alcohol, opposite to what we would expect.

Further, Models 1B-4B show that a lower subjective social status is associated with more a higher likelihood of too little exercise, to smoke, not eating daily fruits or vegetables or not consuming whole wheat bread. For all these health indicators, subjective social status significantly mediates the impact of material class. Furthermore, a lower subjective status is associated with a lower likelihood of too much alcohol and wine consumption, again significantly mediating the impact of material class. When subjective status has an effect on health behavior it as expected explains part of the effect of material class, confirming a mediating effect.

But subjective social status can also play a moderating role in regards to health lifestyle. Models 1D/E-2D/E illustrate how respondents, mainly in the Professional class, are more likely to have too little exercise when subjective status is lower. This shows that the effect of exercising less when having an lower material class is counteracted when their subjective social status is higher since a higher status is consequently associated with more time spent on exercising. Further, Models 5D/E and 6D/E, provide another clear example of the moderating role of subjective social status on the effect of material class since these models illustrate how those in the lower material classes are more likely to consume too much alcohol when their subjective status is lower and consequently less likely to do so when their subjective status is higher. This is highly remarkable since a higher subjective status in itself increases the likelihood of drinking too much while the opposite happens in an interaction. Finally, in regards to healthy food choices, only a small moderating effect is found.

Finally, we conducted four robustness checks to validate our findings. First, we analyzed moderate and vigorous exercise separately and found no substantially different results. Second, in regards to smoking we re-estimated our models by coding those who had smoked in the past as smokers as well. This again did not substantially alter our findings. Third, in regards to alcohol consumption (in general, beer and wine) we re-estimated our models by including consumption not as a dichotomized variable including drinking too much or not, but rather in a linear way. Again, this did not substantially alter our estimates. Finally, we included mild exercise in our index of health lifestyle, and again found no substantial differences. We do not include this measure in our final models because it is not necessarily an indicator of exercise or sports.

## Discussion

In this paper we studied the relationship between material class and health outcomes/health behavior by focusing on two specific mechanisms through which subjective social status plays a role. Following on the Thomas theorem and the finding that people often do not perceive their social position as similar to their material class [26], [27], the implication is that subjective status can explain differences in health and behavior. While earlier studies hinted at an importance of subjective social status in regards to health [5], [21], [22], [60], [85], the pathways through which this works and whether this effect extends to health behaviors has not been examined while experimental studies have shown this to be potentially relevant [48], [49], [50]. In our study we focus on how exactly subjective status plays a role while also focusing on health behavior next to health outcomes.

Regression analyses on a combined dataset of the second (2004–2005) and sixth wave (2012–2013) of the English Longitudinal Study of Ageing and data from the fifth wave (1997–1999) of the Whitehall II survey show that subjective status plays a role in predicting both health outcomes (measured through biomarkers indicating cardiovascular health) and health behavior.

We expected two mechanisms to be at play. Firstly, subjective status was expected to mediate the effect of one’s material class on health and behavior [22]. Secondly, following the logic of [28], subjective status was expected to have a moderating impact by influencing the relationship between material class and health/health behavior. Our results on two indices of health and health behaviors and their respective separate indicators allow us to make several conclusions. First, somewhat remarkably we find no confirmation of the social gradient in regards to material social class when it comes to health biomarkers while it does exist in regards to health lifestyle. Subjective social status on the other hand is important in both cases, in that sense that a lower subjective status is associated with worse health and an unhealthier lifestyle. However, when assessing the separate indicators we can draw two conclusions regarding the social gradient. While research often confirms the social gradient between material class and health outcomes [2], [40], [42] our findings nuance this. While the social gradient occurs in regards to both health outcomes as well as for health behaviors, this does not occur for every health biomarker. Further, while the social gradient exists for all indicators of health lifestyle, this is not always in the expected direction of a higher material class being associated with healthier lifestyle-choices.

Further our findings confirm the relevance of both our expected mechanisms of subjective social status in regards to health outcomes and behavior, although with some differences. Concerning health outcomes, we find that those perceiving their status to be low tend to be unhealthier, regardless of their material class background. When observing the separate models we find a mediating and small moderating effect of subjective status for some biomarkers. This mediating effect of subjective status however, emerges more clearly when it comes to health behavior. A higher subjective status is related to more exercise, lower chances of smoking and less unhealthy eating, which is in line with behavior of the higher material class [86]. What may be remarkable at first sight is that those with a higher material class or higher subjective status are more likely of drinking too much alcohol and wine, while less risk behavior is expected from them [34], [35], [87]. While a higher material class or subjective status is related to a higher likelihood of drinking too much [87] problematic consequences of alcohol consumption are more visible lower in the social stratification [88]. We also find confirmation of a moderating effect when it comes to health lifestyle since on the one hand individuals with a lower material position exercise more when their subjective status is higher. This makes sense because people with a higher social position tend to exercise more [89] and those perceiving themselves as having a higher position want to maintain a positive social identity [51], [52], [55]. In order to do so they follow what they believe is normal for a higher social position [7], [54]. Sports participation has been shown to play a role in peer acceptance [90], so in order to be accepted, individuals within the lower material classes exercise more when their subjective status is higher.

On the other hand, regarding alcohol consumption, another social form of health behavior, the trends found are opposite to what we expected and somewhat more paradoxical at first sight. While people in the higher material class are more likely to consume too much alcohol [87], just as those with a higher subjective status, this trend is exactly the opposite when those with a lower material class have a higher subjective status. Although they are expected to adapt to the behavior of a higher social position [7], they do the opposite by drinking even less (and consequently being less likely to consume too much alcohol) when their subjective social status is higher. While overall, a lower subjective status is associated with a lower chance of drinking too much, it is related to a higher chance of doing so in the lower material classes. This can be explained by the fact that people often think that alcohol abuse occurs more in the lower classes [88], [91] and since people often have a social circle consisting of others with a similar material position [63], [64], this generates the idea for those within the lower classes with a higher subjective status that alcohol consumption all together is negative because they see more alcohol abuse. And as a consequence associate reducing alcohol consumption with behavior fitting to a higher subjective status, while in reality the opposite occurs both in the higher material classes and for those with a higher subjective status.

To summarize, our findings show that the social gradient does not exist for all health biomarkers while it consistently emerges for health lifestyle, thus nuancing the literature on the social gradient anticipating strong class differences regarding health as well [16], [17], [18], [19]. Further subjective social status has some importance for health itself, but has a more prominent mediating and moderating impact on health lifestyle. These different findings regarding health outcomes and health lifestyle may be remarkable but can be explained by the fact that the lifestyle people lead is in essence a choice they make, albeit often in part an unconscious one [10], [65], [92]. Health itself is influenced by this lifestyle people lead [39], [59] but is to a certain extent also a consequence of genetic predispositions as well [29]. While both material class and subjective social status play a role in explaining differences regarding health and health lifestyle, this genetic component of the former can help explain the difference in findings. Our study underscores the existence of substantial differences regarding the social gradient on cardiovascular health and health lifestyle, making it a relevant endeavor for future research to further clarify the explanatory factors causing this difference.

While our two studies extended research on the importance of subjective status from health outcomes to health behavior while at the same time assessing the mechanisms through which this happens, there are some limitations to our approach. First, because of data reasons, we use the Whitehall II Study, conducted amongst British civil servants, to study health behavior. While this study is a very rich source with detailed information on several health related behaviors it is not necessarily representative for the British society, since the participants are all urban, residents of London. Although different types of material class backgrounds are part of the survey, including manual and non-manual workers, it is possible that there are differences with manual and non-manual workers outside of the Civil Service and the city of London. Due to an overrepresentation of non-manual workers, our findings are potentially conservative since unskilled workers or the true underclass are not present. A second limitation of our paper is the fact that again because of data limitations we used two separate studies to look at health and health behavior. While both our data sources are highly qualitative it seems relevant in follow-up research to look at how health and health behavior influence each other since health is a consequence of a cumulative process [16], [18], [19]. Furthermore, using a longitudinal approach towards subjective status and potential changes within it can add to our understanding of health differences. Finally, we are able to control quite extensively for the social background of people by including their class, their subjective identity and their level of education but other confounders such as their parent’s class, ethnicity or religious denomination could play a role. While our data does not allow to include these, it is relevant in future research to see whether these are important.

To conclude, our paper illustrates how subjective status is important for understanding health outcomes and health behavior. The importance of subjective social status in understanding the relationships between material class on the one hand and health or health behavior on the other hand has not often been studied, especially not in the case of health behavior. However, our study shows how subjective social status can help explain differences in both cases since people can be motivated by their social identity to adapt to what they feel as being expected from them. One’s perception of reality and social identity can strongly influence one’s behavior. By further disentangling this relationship between material position and subjective perception, we can better understand the material and cultural processes in health and behavior.

## Supporting information

S1 Table Descriptives of the ELSA and Whitehall II-sample. (PDF)

S2 Table Material class and subjective social status regressed on the separate health biomarkers with control variables. (PDF)

S3 Table Material class and subjective social status regressed on Unhealthy Behaviors including control variables. (PDF)
