# Supplementary material for: Mind over matter. The impact of subjective social status on health outcomes and health behaviors
Source: PLoS One. 2018 Sep 5;13(9):e0202489. doi: 10.1371/journal.pone.0202489 (PMC6124725; doi:10.1371/journal.pone.0202489)
Supplement: S1 Table — (PDF) [file pone.0202489.s001.pdf]

S1 Table. Descriptives of the ELSA and Whitehall II-sample

	Range	Study 1	Study 2
Independent variables			
**Material Class**			
Higher Class (Ref.)	0/1	1345 (37.49%)	2802 (44.65%)
Middle Class	0/1	1242 (34.61%)	2737 (43.62%)
Working Class	0/1	1001 (27.90%)	736 (11.73%)
**Gender**			
Male (Ref.)	0/1	1648 (45.93%)	4538 (72.32%)
Female	0/1	1940 (54.07%)	1737 (27.68%)
**Education**			
No post-secondary education (Ref.)	0/1	2980 (83.05%)	4031 (64.24%)
Post-secondary education	0/1	608 (16.95%)	2244 (35.76%)
**Subjective Social Status Groups**			
Higher Status	0/1	1378 (38.41%)	2048 (32.64%)
Middle Status	0/1	986 (27.48%)	2327 (37.08%)
Lower Status	0/1	1224 (34.11%)	1900 (30.28%)
**Marital Status**			
Married (Ref.)	0/1	2370 (66.05%)	4958 (79.01%)
Unmarried	0/1	1218 (33.95%)	1317 (20.99%)
**Class-Status Combinations**			
Higher class-Higher status (Ref.)	0/1	751 (20.93%)	1596 (25.43%)
Higher class-Middle status	0/1	337 (9.39%)	992 (15.81%)
Higher class-Lower status	0/1	257 (7.16%)	214 (3.41%)
Middle class-Higher status	0/1	405 (11.29%)	416 (6.63%)
Middle class-Middle status	0/1	385 (10.73%)	1179 (18.79%)
Middle class-Lower status	0/1	452 (12.60%)	1142 (18.20%)
Working class-Higher status	0/1	222 (6.19%)	36 (0.57%)
Working class-Middle status	0/1	264 (7.36%)	156 (2.49%)
Working class-Lower status	0/1	515 (14.35%)	544 (8.67%)
**Taking cholesterol medication**			
No (Ref.)	0/1	3310 (92.25)	
Yes	0/1	278 (7.75)	
**Age**	60-90/45-69/	71.51 (7.88)	55.78 (6.01)
**Subjective Status**	100-5/10-0.5	42.09 (16.33)	4.58 (1.61)
Dependent variables			
**Health**	0-5	1.76 (1.04)	
**Health lifestyle**	0-7		2.506 (1.07)
**Too little exercise/week**			
No (Ref.)	0/1		525 (8.37%)
Yes	0/1		5750 (91.63%)
**Too much alcohol/week**			
No (Ref.)	0/1		4640 (74.50%)
Yes	0/1		1588 (25.50%)
**Too much wine/week**			
No (Ref.)	0/1		5940 (95.38%)
Yes	0/1		288 (4.62%)
**Too much beer/week**			
No (Ref.)	0/1		6023 (96.71%)
Yes	0/1		205 (3.29%)

**Eating whole wheat bread**			
No (Ref.)	0/1		2373 (38.32%)
Yes	0/1		3819 (61.68%)
**Eating daily fruits and vegetables**			
No (Ref.)	0/1		4604 (74.10%)
Yes	0/1		1609 (25.90%)
**Smoking**			
No (Ref.)	0/1		5621 (90.02%)
Yes	0/1		623 (9.98%)
**Systolic blood pressure**			
Not too high (Ref.)	0/1	1474 (42.75%)	
Too high	0/1	1974 (57.25%)	
**Diastolic blood pressure**			
Not too high (Ref.)	0/1	2017 (58.50%)	
Too high	0/1	1431 (41.50%)	
**Cholesterol**			
Not too high (Ref.)	0/1	1786 (64.45%)	
Too high	0/1	985 (35.55%)	
**Triglycerides**			
Not too high (Ref.)	0/1	2385 (88.04%)	
Too high	0/1	324 (11.96%)	
**High sensitivity c-reactive protein**			
Not too high (Ref.)	0/1	1965 (72.51%)	
Too high	0/1	745 (27.49%)	
**HDL-cholesterol**			
Not too high (Ref.)	0/1	2518 (92.25%)	
Too high	0/1	191 (7.05%)	

Source: Wave 2 and 6 of the English Longitudinal Study of Ageing & Wave 5 of the Whitehall II Study
