# Supplementary material for: Mind over matter. The impact of subjective social status on health outcomes and health behaviors
Source: PLoS One. 2018 Sep 5;13(9):e0202489. doi: 10.1371/journal.pone.0202489 (PMC6124725; doi:10.1371/journal.pone.0202489)
Supplement: S2 Table — (PDF) [file pone.0202489.s002.pdf]

Confirmatory factor analysis on our index of health biomarkers shows that not all variables load in a single factor nor is there a clear structure of several underlying factors, except for the two measurements of blood pressure loading similarly, while the Cronbach's alpha is 0.48. Since this illustrates that the several health biomarkers are not strongly related to each other, we present the following separate analyses on each of the biomarkers to further show how material class and subjective social status are associated with health outcomes.

S2 Table. Material Class and Subjective Social Status regressed on the separate health biomarkers with control variables

**Systolic blood pressure** **(N: 3448)**	1A	1B	1C	1D	1E
**Constant**	0.020 (0.01)***	0.015 (0.01)***	0.019 (0.01)***	0.036 (0.02)***	0.019 (0.01)***
**Material Class**					
Higher class (Ref.)	-	-	-	-	
Middle class	1.016 (0.09)	0.983 (0.09)	0.987 (0.09)	0.740 (0.27)	
Working class	1.193 (0.12)*	1.115 (0.11)	1.131 (0.11)	1.181 (0.42)	
**Gender**					
Male (Ref.)	-	-	-	-	-
Female	0.745 (0.06)***	0.750 (0.06)***	0.749 (0.06)***	0.747 (0.06)**	0.743 (0.06)***
**Education**					
Lower education (Ref.)	-	-	-	-	-
Post-secondary education	0.780 (0.08)*	0.826 (0.09)	0.812 (0.09)*	0.824 (0.09)	0.811 (0.09)*
**Marital Status**					
Married (Ref.)	-	-	-	-	-
Not-married	1.111 (0.09)	1.075 (0.09)	1.087 (0.09)	1.075 (0.08)	1.083 (0.09)
**Age**	1.063 (0.00)***	1.063 (0.01)***	1.062 (0.01)***	1.063 (0.01)***	1.062 (0.01)***
**Decreasing Subjective Status**		1.007 (0.00)**		1.008 (0.00)*	
**Subjective Status Groups**					
Higher status (Ref.)			-		
Middle status			1.097 (0.10)		
Lower status			1.231 (0.11)*		
**Material Class X Subjective Status**					
Higher X Subj status (Ref.)				-	
Middle X Subj Status				0.995 (0.01)	
Working X Subj Status				1.001 (0.01)	
**Class-Status Combinations**					
Higher class-Higher status (Ref.)					-
Higher class-Middle status					1.051 (0.15)
Higher class-Lower status					1.452 (0.23)*
Middle class-Higher status					1.104 (0.15)
Middle class-Middle status					1.178 (0.17)
Middle class-Lower status					1.074 (0.14)
Working class-Higher status					1.036 (0.17)
Working class-Middle status					1.249 (0.20)

Working class-Lower status					1.507 (0.20)**
**Karlson, Holm, Breen-Mediation Analysis**					
**% of Material social class mediated by Subjective social status**					
Higher Class		-	-		
Middle Class		210.54%*	187.99%*		
Working Class		38.51%**	29.76%*		
**Diastolic blood pressure** **(N:3448)**	2A	2B	2C	2D	2E
**Constant**	0.024 (0.01)***	0.037 (0.01)***	0.023 (0.01)***	0.040 (0.02)***	0.023 (0.01)***
**Material Class**					
Higher class (Ref.)	-	-	-	-	
Middle class	1.038 (0.09)	1.017 (0.09)	1.020 (0.09)	0.735 (0.27)	
Working class	1.280 (0.12)**	1.227 (0.12)*	1.235 (0.12)*	0.862 (0.30)	
**Gender**					
Male (Ref.)	-	-	-	-	-
Female	0.733 (0.05)***	0.738 0.05)***	0.738 (0.05)***	0.733 (0.05)***	0.733 (0.05)***
**Education**					
Lower education (Ref.)	-	-	-	-	-
Post-secondary education	0916 (0.10)	0.950 (0.10)	0.936 (0.10)	0.966 (0.11)	0.944 (0.10)
**Marital Status**					
Married (Ref.)	-	-	-	-	-
Not-married	1.215 (0.10)*	1.191 (0.09)*	1.197 (0.10)*	1.194 (0.10)*	1.194 (0.10)*
**Age**	1.049 (0.00)***	1.049 (0.00)***	1.049 (0.00)***	1.049 (0.00)***	1.049 0.00)***
**Decreasing Subjective Status**		1.004 (0.00)		1.008 (0.00)	
**Subjective Status Groups**					
Higher status (Ref.)			-		
Middle status			1.010 (0.09)		
Lower status			1.144 (0.10)		
**Material Class X Subjective Status**					
Higher X Subj status (Ref.)				-	
Middle X Subj Status				0.995 (0.01)	
Working X Subj Status				0.994 (0.01)	
**Class-Status Combinations**					
Higher class-Higher status (Ref.)					-
Higher class-Middle status					0.946 (0.14)
Higher class-Lower status					1.442 (0.23)*
Middle class-Higher status					1.122 (0.15)
Middle class-Middle status					1.145 (0.16)
Middle class-Lower status					1.074 (0.15)
Working class-Higher status					1.249 (0.21)
Working class-Middle status					1.313 (0.21)
Working class-Lower status					1.464 (0.19)**
**Karlson, Holm, Breen-Mediation Analysis**					

**% of Material social class mediated by Subjective social status**					
Higher Class		-	-		
Middle Class		54.48%	50.13%		
Working Class		17.05%	13.75%		
**Cholesterol (N: 2771)**	3A	3B	3C	3E	3E
**Constant**	2.218 (0.90)*	2.474 (1.03)*	2.272 (0.92)*	2.417 (1.05)*	1.850 (0.80)
**Material Class**					
Higher class (Ref.)	-	-	-	-	
Middle class	0.963 (0.10)	0.978 (0.10)	0.983 (0.10)	0.961 (0.41)	
Working class	0.898 (0.10)	0.930 (0.11)	0.929 (0.11)	0.840 (0.34)	
**Gender**					
Male (Ref.)	-	-	-	-	-
Female	2.391 (0.21)***	2.387 0.21)***	2.39 (0.21)***	2.386 (0.21)***	2.385 (0.21)***
**Education**					
Lower education (Ref.)	-	-	-	-	-
Post-secondary education	1.017 (0.12)	0.990 (0.12)	0.987 (0.12)	0.995 (0.12)	1.000 (0.12)
**Marital Status**					
Married (Ref.)	-	-	-	-	-
Not-married	0.833 (0.08) *	0.846 (0.08)	0.847 (0.07)	0.847 (0.07)	0.847 (0.08)
**Age**	0.975 (0.01)***	0.975 (0.01)***	0.976 (0.01)***	0.975 (0.01)***	0.975 (0.00)***
**Decreasing Subjective Status**		0.997 (0.00))		0.997 (0.00)	
**Subjective Status Groups**					
Higher status (Ref.)			-		
Middle status			0.877 (0.09)		
Lower status			0.878 (0.09)		
**Material Class X Subjective Status**					
Higher X Subj status (Ref.)				-	
Middle X Subj Status				1.000 (0.01)	
Working X Subj Status				1.002 (0.01)	
**Class-Status Combinations**					
Higher class-Higher status (Ref.)					-
Higher class-Middle status					1.024 (0.16)
Higher class-Lower status					0.968 (0.18)
Middle class-Higher status					1.098 (0.17)
Middle class-Middle status					0.844 (0.14)
Middle class-Lower status					0.936 (0.15)
Working class-Higher status					1.084 (0.21)
Working class-Middle status					0.858 (0.16)
Working class-Lower status					0.837 (0.13)
**Karlson, Holm, Breen-Mediation Analysis**					
**% of Material social class mediated by Subjective social status**					
Higher Class		-	-		
Middle Class		43.02%	52.22%		
Working Class		32.48%	34.07%		

**Triglycerides** **(N: 2709)**	4A	4B	4C	4D	4E
**Constant**	0.655 (0.37)	0.289 (0.18)*	0.384 (0.23)	0.230 (0.15)*	0.360 (0.22)
**Material Class**					
Higher class (Ref.)	-	-	-	-	
Middle class	1.138 (0.17)	1.080 (0.16)	1.078 (0.16)	1.671 (0.67)	
Working class	1.017 (0.17)	0.914 (0.15)	0.922 (0.16)	1.283 (0.59)	
**Gender**					
Male (Ref.)	-	-	-	-	-
Female	0.798 (0.10)*	0.807 (0.10)	0.807 (0.10)	0.798 (0.10)	0.796 (0.10)
**Education**					
Lower education (Ref.)	-	-	-	-	-
Post-secondary education	0.635 (0.12)*	0.691 (0.13)	0.675 0.13)*	0.710 (0.14)	1.341 (0.27)
**Marital Status**					
Married (Ref.)	-	-	-	-	-
Not-married	1.074 (0.14)	1.022 (0.14)	1.032 (0.14)	1.022 (0.14)	1.028 (0.14)
**Cholesterol medication**					
No (Ref)	-	-	-	-	-
Yes	1.337 (0.27)	1.351 (0.27)	1.344 (0.27)	1.363 (0.27)	1.341 (0.27)
**Age**	0.986 (0.01)	0.985 (0.01)	0.985 (0.01)	0.985 (0.01)	0.985 (0.01)
**Decreasing Subjective Status**		1.010 (0.00)*		1.017 (0.01)*	
**Subjective Status Groups**					
Higher status (Ref.)			-		
Middle status			1.109 (0.18)		
Lower status			1.412 (0.21)*		
**Material Class X Subjective Status**					
Higher X Subj status (Ref.)				-	
Middle X Subj Status				0.989 (0.01)	
Working X Subj Status				0.992 (0.01)	
**Class-Status Combinations**					
Higher class-Higher status (Ref.)					-
Higher class-Middle status					1.019 (0.27)
Higher class-Lower status					2.072 (0.51)**
Middle class-Higher status					1.337 (0.31)
Middle class-Middle status					1.345 (0.32)
Middle class-Lower status					1.451 (0.33)
Working class-Higher status					0.948 (0.30)
Working class-Middle status					1.272 (0.35)
Working class-Lower status					1.359 (0.31)
**Karlson, Holm, Breen-Mediation Analysis**					
**% of Material social class mediated by Subjective social status**					
Higher Class		-	-		
Middle Class		38.16%*	41.58%		
Working Class		813.26%*	622.26%		
**High sensitivity c-reactive protein** **(N: 2710)**	5A	5B	5C	5D	5E

**Constant**	0.0 (0.04)***	0.124 (0.06)***	0.085 (0.04)***	0.062 (0.03)***	0.084 (0.04)***
**Material Class**					
Higher class (Ref.)	-	-	-	-	
Middle class	1.207 (0.13)	1.177 (0.13)	1.182 (0.13)	1.647 (0.49)	
Working class	1.342 (0.16)*	1.271 (0.15)*	1.291 (0.16)*	1.574 (0.51)	
**Gender**					
Male (Ref.)	-	-	-	-	-
Female	1.197 (0.11)*	-1.204 (0.11)*	1.201 (0.11)*	1.194 (0.11)	1.198 (0.11)*
**Education**					
Lower education (Ref.)	-	-	-	-	-
Post-secondary education	0.641 (0.09)**	0.670 (0.10)**	0.658 (0.09)**	0.681 (0.10)**	0.667 (0.10)**
**Marital Status**					
Married (Ref.)	-	-	-	-	-
Not-married	1.154 (0.11)	1.125 (0.11)	1.137 (0.11)	1.125 (0.11)	1.133 (0.11)
**Cholesterol medication**					
No (Ref)	-	-	-	-	-
Yes	0.970 (0.16)	0.976 (0.16)	0.974 (0.16)	0.982 (0.16)	0.667 (0.10)**
**Age**	1.018 (0.01) **	1.017 (0.01)**	1.017 (0.01)**	1.018 (0.01)**	1.017 (0.01)**
**Decreasing Subjective Status**		1.005 (0.00)		1.010 (0.01)*	
**Subjective Status Groups**					
Higher status (Ref.)			-		
Middle status			1.071 (0.12)		
Lower status			1.153 (0.13)		
**Material Class X Subjective Status**					
Higher X Subj status (Ref.)				-	
Middle X Subj Status				0.991 (0.01)	
Working X Subj Status				0.994 (0.01)	
**Class-Status Combinations**					
Higher class-Higher status (Ref.)					-
Higher class-Middle status					0.880 (0.17)
Higher class-Lower status					1.433 (0.28)
Middle class-Higher status					1.159 0.20)
Middle class-Middle status					1.411 (0.24)*
Middle class-Lower status					1.262 (0.21)
Working class-Higher status					1.321 (0.28)
Working class-Middle status					1.452 (0.28)*
Working class-Lower status					1.443 (0.23)*
**Karlson, Holm, Breen-Mediation Analysis**					
**% of Material social class mediated by Subjective social status**					
Higher Class		-	-		
Middle Class		13.55%	11.32%		
Working Class		18.60%	13.30%		
**HDL-cholesterol (N: 2709)**	6A	6B	6C	6D	6E
**Constant**	0.158 (0.12) *	0.102 (0.08)	0.154 (0.12)*	0.076 (0.06)	0.141 (0.11)
**Material Class**					

Higher class (Ref.)	-	-	-	-	
Middle class	1.277 (0.24)	1.164 (0.22)	1.192 (0.23)	2.329 (1.20)	
Working class	1.161 (0.25)	0.987 (0.22)	1.041 (0.23)	1.386 (0.86)	
**Gender**					
Male (Ref.)	-	-	-	-	-
Female	0.181 (0.03)***	0.183 (0.03)***	0.183 (0.03)***	0.181 (0.03)***	0.179 (0.03)***
**Education**					
Lower education (Ref.)	-	-	-	-	-
Post-secondary education	0.622 (0.15)*	0.708 (0.17)	0.667 (0.16)	0.734 (0.18)	0.691 (0.17)
**Marital Status**					
Married (Ref.)	-	-	-	-	-
Not-married	1.282 (0.22)	1.200 (0.21)	1.231 (0.21)	1.195 (0.21)	1.226 (0.21)
**Cholesterol medication**					
No (Ref)	-	-	-	-	-
Yes	1.830 (0.43)*	1.873 (0.44)	1.84 (0.43)***	1.885 (0.44)**	1.815 (0.43)*
**Age**	0.996 (0.01)	0.995 (0.01)	0.995 (0.01)	0.977 (0.01)**	0.994 (0.01)
**Decreasing Subjective Status**		1.014 (0.01)**		1.023 (0.01)**	
**Subjective Status Groups**					
Higher status (Ref.)			-		
Middle status			1.120 (0.23)		
Lower status			1.448 (0.28)		
**Material Class X Subjective Status**					
Higher X Subj status (Ref.)				-	
Middle X Subj Status				0.983 (0.01)	
Working X Subj Status				0.991 (0.01)	
**Class-Status Combinations**					
Higher class-Higher status (Ref.)					-
Higher class-Middle status					1.184 (0.39)
Higher class-Lower status					2.417 (0.75)**
Middle class-Higher status					1.840 (0.53)*
Middle class-Middle status					1.402 (0.45)
Middle class-Lower status					1.754 (0.50)*
Working class-Higher status					0.987 (0.44)
Working class-Middle status					1.814 (0.64)
Working class-Lower status					1.647 (0.49)
**Karlson, Holm, Breen-Mediation Analysis**					
**% of Material social class mediated by Subjective social status**					
Higher Class		-	-		
Middle Class		30.78%*	24.44%		
Working Class		110.20%**	71.34%		

Source: Wave 2 and 6 of the English Longitudinal Study of Ageing ° p > 0.10 * p < 0.05, ** p < 0.01, *** p < 0.001. Entries represent the results of separate logistic regression models
