# Supplementary material for: Mind over matter. The impact of subjective social status on health outcomes and health behaviors
Source: PLoS One. 2018 Sep 5;13(9):e0202489. doi: 10.1371/journal.pone.0202489 (PMC6124725; doi:10.1371/journal.pone.0202489)
Supplement: S3 Table — (PDF) [file pone.0202489.s003.pdf]

As was the case in Study 1, confirmatory factor analysis on our index of unhealthy behavior shows that not all variables load in a single factor nor is there a clear structure of several underlying factors while the Cronbach's alpha is low at 0.20. Since this shows that the several forms of unhealthy behavior are not very related to each other, we present the following separate analyses further showing how material class and subjective social status are associated with several forms of behavior.

**S3 Table. Material Class and Subjective Social Status regressed on Unhealthy Behaviors including control variables**

| ***Too little exercise*** ***(N: 6275)*** | 1A | 1B | 1C | 1D | 1E |
|---|---|---|---|---|---|
| **Constant** | 23.492 (10.38)*** | 13.311 (6.30)*** | 21.102 (9.61)*** | 44.526 (26.69)*** | 22.618 (10.36)*** |
| **Material Class** | | | | | |
| Administrative Class (Ref.) | - | - | - | - | |
| Professional/Executive Class | 1.281 (0.13)* | 1.014 (0.12) | 1.020 (0.12) | 0.436 (0.15)* | |
| Clerical/Support Class | 1.868 (0.39)** | 1.236 (0.28) | 1.289 (0.29) | 1.344 (1.108) | |
| **Gender** | | | | | |
| Male (Ref.) | - | - | - | - | - |
| Female | 1.874 (0.25)*** | 1.918 (0.26)*** | 1.905 (0.26)*** | 1.904 (0.26)*** | 1.892 (0.26)*** |
| **Education** | | | | | |
| No post-secondary education (Ref.) | - | - | - | - | - |
| Post-secondary education | 1.235 (0.13)* | 1.349 (0.14)** | 1.336 (0.14)** | 1.307 (0.14)* | 1.315 (0.14)* |
| **Marital Status** | | | | | |
| Married (Ref.) | - | - | - | - | - |
| Not-married | 1.219 (0.16) | 1.152 (0.15) | 1.165 (0.15) | 1.148 (0.15) | 1.159 (0.15) |
| **Age** | 0.979 (0.01)** | 0.978 (0.01)** | 0.978 (0.01)** | 0.978 (0.01)** | 0.978 (0.01)** |
| **Decreasing Subjective Status** | | 1.188 (0.04)*** | | 1.071 (0.05) | |
| **Subjective Status Groups** | | | | | |
| Higher status (Ref.) | | | - | | |
| Middle status | | | 1.434 (0.16)** | | |
| Lower status | | | 1.903 (0.28)*** | | |
| **Material Class X Subjective Status** | | | | | |
| Administrative X Subj status (Ref.) | | | | - | |
| Professional/Executive X Subj Status | | | | 1.224 (0.09)** | |
| Clerical/Support X Subj Status | | | | 1.025 (0.14) | |
| **Class-Status Combinations** | | | | | |
| Administrative-Higher status (Ref.) | | | | | - |
| Administrative-Middle status | | | | | 1.391 (0.20)* |
| Administrative-Lower status | | | | | 0.970 (0.22) |
| Professional-Higher status | | | | | 0.769 (0.13) |
| Professional-Middle status | | | | | 1.385 (0.20)* |
| Professional-Lower status | | | | | 2.128 (0.35)*** |
| Clerical-Higher status | | | | | 3.015 (3.09) |
| Clerical-Middle status | | | | | 1.294 (0.44) |
| Clerical-Lower status | | | | | 2.463 (0.63)*** |

| | | | | | |
|---|---|---|---|---|---|
| **% of Material social class mediated by Subjective social status** | | | | | |
| Higher Class | | - | - | | |
| Middle Class | | 94.26%* | 91.36%** | | |
| Working Class | | 64.47%** | 58.25*%** | | |
| ***No consumption of whole wheat bread*** ***(N: 6405)*** | 2A | 2B | 2C | 2D | 2E |
| **Constant** | 2.079 (0.53)** | 1.698 (0.46)* | 1.957 (0.51)* | 2.237 (0.78)* | 1.985 (0.52)** |
| **Material Class** | | | | | |
| Administrative Class (Ref.) | - | - | - | - | |
| Professional/Executive Class | 1.203 (0.07)** | 1.141 (0.08)* | 1.173 (0.08)* | 0.927 (0.18) | |
| Clerical/Support Class | 1.425 (0.14)*** | 1.281 (0.14)* | 1.344 (0.15)** | 1.233 (0.47) | |
| **Gender** | | | | | |
| Male (Ref.) | - | - | - | - | - |
| Female | 0.748 (0.05)*** | 0.757 (0.05)*** | 0.755 (0.05)*** | 0.756 (0.05)*** | 0.753 (0.05)*** |
| **Education** | | | | | |
| No post-secondary education (Ref.) | - | - | - | - | - |
| Post-secondary education | 0.809 (0.05)*** | 0.829 (0.05)** | 0.819 (0.05)** | 0.824 (0.05)** | 0.817 (0.05)** |
| **Marital Status** | | | | | |
| Married (Ref.) | - | - | - | - | - |
| Not-married | 0.830 (0.05)** | 0.818 (0.05)** | 0.826 (0.06)** | 0.817 (0.05)** | 0.826 (0.06)** |
| **Age** | 0.997 (0.00) | 0.998 (0.00) | 0.997 (0.00) | 0.997 (0.00) | 0.997 (0.00) |
| **Decreasing Subjective Status** | | 1.042 (0.02)* | | 1.016 (0.03) | |
| **Subjective Status Groups** | | | | | |
| Higher status (Ref.) | | | - | | |
| Middle status | | | 1.029 (0.07) | | |
| Lower status | | | 1.083 (0.09) | | |
| **Material Class X Subjective Status** | | | | | |
| Administrative X Subj status (Ref.) | | | | - | |
| Professional/Executive X Subj Status | | | | 1.050 (0.05) | |
| Clerical/Support X Subj Status | | | | 1.017 (0.07) | |
| **Class-Status Combinations** | | | | | |
| Administrative-Higher status (Ref.) | | | | | - |
| Administrative-Middle status | | | | | 0.996 (0.08) |
| Administrative-Lower status | | | | | 1.109 (0.17) |
| Professional-Higher status | | | | | 1.087 (0.13) |
| Professional-Middle status | | | | | 1.218 (0.10)* |
| Professional-Lower status | | | | | 1.263 (0.11)** |
| Clerical-Higher status | | | | | 1.919 (0.74) |
| Clerical-Middle status | | | | | 1.377 (0.26) |
| Clerical-Lower status | | | | | 1.406 (0.17)** |

| | | | | | |
|---|---|---|---|---|---|
| **% of Material social class mediated by Subjective social status** | | | | | |
| Higher Class | | - | - | | |
| Middle Class | | 28.83%* | 14.03% | | |
| Working Class | | 27.16%* | 12.19% | | |

| ***No daily consumption of fruits and vegetables*** ***(N: 6430)*** | 3A | 3B | 3C | 3D | 3E |
|---|---|---|---|---|---|
| **Constant** | 3.847 (1.11)*** | 1.893 (0.58)* | 3.268 (0.97)*** | 11.837 (4.77)*** | 3.236 (0.97)*** |
| **Material Class** | | | | | |
| Administrative Class (Ref.) | - | - | - | - | |
| Professional/Executive Class | 1.435 (0.10)*** | 1.106 (0.08) | 1.124 (0.09) | 0.842 (0.20) | |
| Clerical/Support Class | 2.734 (0.30)*** | 1.772 (0.22)*** | 1.855 (0.22)*** | 2.222 (0.91)* | |
| **Gender** | | | | | |
| Male (Ref.) | - | - | - | - | - |
| Female | 0.436 (0.03)*** | 0.442 (0.03)*** | 0.436 (0.03)*** | 0.440 (0.03)*** | 0.432 (0.03)*** |
| **Education** | | | | | |
| No post-secondary education (Ref.) | - | - | - | - | - |
| Post-secondary education | 0.756 (0.05)*** | 0.820 (0.06)** | 0.806 (0.06)** | 0.815 (0.06)** | 0.809 (0.06)** |
| **Marital Status** | | | | | |
| Married (Ref.) | - | - | - | - | - |
| Not-married | 1.450 (0.10)*** | 1.349 (0.10)*** | 1.372 (0.10)*** | 1.349 (0.10)*** | 1.367 (0.10)*** |
| **Age** | 0.957 0.00)*** | 0.957 (0.00)*** | 0.956 (0.00)*** | 0.957 (0.00)*** | 0.957 (0.00)*** |
| **Decreasing Subjective Status** | | 1.200 (0.03)*** | | 1.145 (0.03)*** | |
| **Subjective Status Groups** | | | | | |
| Higher status (Ref.) | | | - | | |
| Middle status | | | 1.292 (0.10)** | | |
| Lower status | | | 1.959 (0.18)*** | | |
| **Material Class X Subjective Status** | | | | | |
| Administrative X Subj status (Ref.) | | | | - | |
| Professional/Executive X Subj Status | | | | 1.062 (0.05) | |
| Clerical/Support X Subj Status | | | | 0.974 (0.07) | |
| **Class-Status Combinations** | | | | | |
| Administrative-Higher status (Ref.) | | | | | - |
| Administrative-Middle status | | | | | 1.415 (0.14)** |
| Administrative-Lower status | | | | | 1.556 (0.27)* |
| Professional-Higher status | | | | | 1.116 (0.16) |
| Professional-Middle status | | | | | 1.394 (0.14)** |
| Professional-Lower status | | | | | 2.372 (0.23)*** |
| Clerical-Higher status | | | | | 3.822 (1.42)*** |
| Clerical-Middle status | | | | | 2.334 0.50)*** |
| Clerical-Lower status | | | | | 3.604 (0.47)*** |

| **Karlson, Holm, Breen-Mediation Analysis** | | | | | |
|---|---|---|---|---|---|
| **% of Material social class mediated by Subjective social status** | | | | | |

| | | | | | |
|---|---|---|---|---|---|
| Higher Class<br>Middle Class<br>Working Class | | -<br>70.29%***<br>41.55%*** | -<br>66.26%***<br>36.01%*** | | |
| ***Smoking** (N: 6467)* | 4A | 4B | 4C | 4D | 4E |
| **Constant** | 0.454 (0.19) | 0.283 (0.12)** | 0.362 (0.16)* | 1.009 (0.62) | 0.363 (0.16)* |
| **Material Class** | | | | | |
| Administrative Class (Ref.) | - | - | - | - | |
| Professional/Executive Class | 1.510 0.16)*** | 1.252 (0.14)* | 1.254 (0.14)* | 1.286 (0.45) | |
| Clerical/Support Class | 2.403 (0.35)*** | 1.837 (0.30)*** | 1.939 (0.31)*** | 1.288 (0.68) | |
| **Gender** | | | | | |
| Male (Ref.) | - | - | - | - | - |
| Female | 0.910 (0.09) | 0.951 (0.10) | 0.942 (0.10) | 0.954 (0.09) | 0.944 (0.10) |
| **Education** | | | | | |
| No post-secondary education (Ref.) | - | - | - | - | - |
| Post-secondary education | 0.579 (0.06)*** | 0.616 (0.07)*** | 0.621 (0.07)*** | 0.614 (0.09)*** | 0.621 (0.07)*** |
| **Marital Status** | | | | | |
| Married (Ref.) | - | - | - | - | - |
| Not-married | 1.580 (0.15)*** | 1.509 (0.15)*** | 1.537 (0.15)*** | 1.507 (0.15)*** | 1.538 (0.15)*** |
| **Age** | 0.970 (0.01)*** | 0.970 (0.01)*** | 0.970 (0.01)*** | 0.970 (0.01)*** | 0.971 (0.01)*** |
| **Decreasing Subjective Status** | | 1.131 (0.03)*** | | 1.106 (0.06) | |
| **Subjective Status Groups** | | | | | |
| Higher status (Ref.) | | | - | | |
| Middle status | | | 1.445 (0.18)** | | |
| Lower status | | | 1.642 (0.23)*** | | |
| **Material Class X Subjective Status** | | | | | |
| Administrative X Subj status (Ref.) | | | | - | |
| Professional/Executive X Subj Status | | | | 0.998 (0.07) | |
| Clerical/Support X Subj Status | | | | 1.062 (0.10) | |
| **Class-Status Combinations** | | | | | |
| Administrative-Higher status (Ref.) | | | | | - |
| Administrative-Middle status | | | | | 1.441 (0.24)* |
| Administrative-Lower status | | | | | 1.609 (0.43) |
| Professional-Higher status | | | | | 1.286 (0.28) |
| Professional-Middle status | | | | | 1.816 (0.28)*** |
| Professional-Lower status | | | | | 2.027 (0.32)*** |
| Clerical-Higher status | | | | | 1.364 (0.85) |
| Clerical-Middle status | | | | | 2.693 (0.71)*** |
| Clerical-Lower status | | | | | 3.240 (0.58)*** |
| **Karlson, Holm, Breen-Mediation Analysis** | | | | | |
| **% of Material social class mediated by Subjective social status** | | | | | |
| Higher Class | | - | - | | |
| Middle Class | | 41.68%*** | 39.53%** | | |
| Working Class | | 31.19%*** | 27.00%** | | |

| ***Alcohol Consumption** (N: 6429)* | 5A | 5B | 5C | 5D | 5E |
|---|---|---|---|---|---|
| **Constant** | 2.702 (0.79)** | 4.673 (1.46)*** | 3.612 (1.09)*** | 0.938 (0.37) | 3.476 (1.05)*** |
| **Material Class** | | | | | |
| Administrative Class (Ref.) | - | - | - | - | |
| Professional/Executive Class | 0.763 (0.05)*** | 0.885 (0.06) | 0.862 (0.06)* | 0.642 (0.14)* | |
| Clerical/Support Class | 0.354 (0.05)*** | 0.474 (0.08)*** | 0.431 (0.07)*** | 0.138 (0.09)** | |
| **Gender** | | | | | |
| Male (Ref.) | - | - | - | - | - |
| Female | 0.308(0.03)*** | 0.301 (0.03)*** | 0.303 (0.03)*** | 0.303 (0.03)*** | 0.303 (0.03)*** |
| **Education** | | | | | |
| No post-secondary education (Ref.) | - | - | - | - | - |
| Post-secondary education | 1.008 (0.07) | 0.949 (0.06) | 0.950 (0.06) | 0.933 (0.06) | 0.958 (0.07) |
| **Marital Status** | | | | | |
| Married (Ref.) | - | - | - | - | - |
| Not-married | 1.184 (0.09)* | 1.226 (0.10)* | 1.205 (0.10)* | 1.221 (0.10)* | 1.204 (0.10)* |
| **Age** | 0.970 (0.00)*** | 0.968 (0.00)*** | 0.968 (0.00)*** | 0.968 (0.00)*** | 0.968 (0.00)*** |
| **Decreasing Subjective Status** | | 0.894 (0.02)*** | | 0.821 (0.04)*** | |
| **Subjective Status Groups** | | | | | |
| Higher status (Ref.) | | | - | | |
| Middle status | | | 0.740 (0.05)*** | | |
| Lower status | | | 0.717 (0.07)*** | | |
| **Material Class X Subjective Status** | | | | | |
| Administrative X Subj status (Ref.) | | | | - | |
| Professional/Executive X Subj Status | | | | 1.082 (0.05) | |
| Clerical/Support X Subj Status | | | | 1.249 (0.13)* | |
| **Class-Status Combinations** | | | | | |
| Administrative-Higher status (Ref.) | | | | | - |
| Administrative-Middle status | | | | | 0.806 (0.07)* |
| Administrative-Lower status | | | | | 0.711 (0.12)* |
| Professional-Higher status | | | | | 1.001 (0.13) |
| Professional-Middle status | | | | | 0.632 (0.06)*** |
| Professional-Lower status | | | | | 0.632 (0.06)*** |
| Clerical-Higher status | | | | | 0.416 (0.26) |
| Clerical-Middle status | | | | | 0.247 (0.08)*** |
| Clerical-Lower status | | | | | 0.343 (0.06)*** |
| **Karlson, Holm, Breen-Mediation Analysis** | | | | | |
| **% of Material social class mediated by Subjective social status** | | | | | |
| Higher Class | | - | - | | |
| Middle Class | | 54.37%*** | 44.50%*** | | |
| Working Class | | 24.94%*** | 18.75%*** | | |

| ***Wine Consumption*** ***(N: 6429)*** | 6A | 6B | 6B | 6D | 6E |
|---|---|---|---|---|---|
| **Constant** | 0.096 (0.06)*** | 0.360 (0.23) | 0.162 (0.10)** | 0.007 (0.01)*** | 0.163 (0.10)** |
| **Material Class** | | | | | |
| Administrative Class (Ref.) | - | - | - | - | |
| Professional/Executive Class | 0.470 (0.07)*** | 0.687 (0.11)* | 0.618 (0.10)** | 0.270 (0.13)** | |
| Clerical/Support Class | 0.124 (0.05)*** | 0.242 (0.10)** | 0.187 (0.08)*** | 0.009 (0.2)** | |
| **Gender** | | | | | |
| Male (Ref.) | - | - | - | - | - |
| Female | 1.760 (0.25)*** | 1.656 (0.24)** | 1.663 (0.25)** | 1.660 (0.25)** | 1.669 (0.24)** |
| **Education** | | | | | |
| No post-secondary education (Ref.) | - | - | - | - | - |
| Post-secondary education | 1.725 (0.23)*** | 1.424 (0.20)* | 1.492 (0.21)** | 1.375 (0.19)* | 1.493 (0.21)** |
| **Marital Status** | | | | | |
| Married (Ref.) | - | - | - | - | - |
| Not-married | 0.781 (0.13) | 0.900 (0.15) | 0.865 (0.15) | 0.888 (0.15) | 0.862 (0.15) |
| **Age** | 0.989 (0.01) | 0.984 (0.01) | 0.984 (0.01) | 0.983 (0.01) | 0.984 (0.01) |
| **Decreasing Subjective Status** | | 0.750 (0.04)*** | | 0.504 (0.11)*** | |
| **Subjective Status Groups** | | | | | |
| Higher status (Ref.) | | | - | | |
| Middle status | | | 0.578 (0.09)*** | | |
| Lower status | | | 0.506 (0.10)** | | |
| **Material Class X Subjective Status** | | | | | |
| Administrative X Subj status (Ref.) | | | | - | |
| Professional/Executive X Subj Status | | | | 1.265 (0.14)* | |
| Clerical/Support X Subj Status | | | | 1.834 (0.51)* | |
| **Class-Status Combinations** | | | | | |
| Administrative-Higher status (Ref.) | | | | | - |
| Administrative-Middle status | | | | | 0.596 (0.10)** |
| Administrative-Lower status | | | | | 0.329 (0.14)** |
| Professional-Higher status | | | | | 0.598 (0.15)* |
| Professional-Middle status | | | | | 0.351 (0.07)*** |
| Professional-Lower status | | | | | 0.318 (0.07)*** |
| Clerical-Higher status | | | | | - |
| Clerical-Middle status | | | | | - |
| Clerical-Lower status | | | | | 0.139 (0.06)*** |
| **Karlson, Holm, Breen-Mediation Analysis** | | | | | |
| **% of Material social class mediated by Subjective social status** | | | | | |
| Higher Class | | - | - | | |
| Middle Class | | 49.39%*** | 35.26%*** | | |
| Working Class | | 31.00%*** | 20.18%*** | | |
| ***Beer Consumption*** ***(N: 6429)*** | 7A | 7B | 7C | 7D | 7E |

| | | | | | |
|---|---|---|---|---|---|
| **Constant** | 1.744 (1.28) | 1.896 (1.48) | 1.692 (1.28) | 1.361 (1.39) | 1.666 (1.27) |
| **Material Class** | | | | | |
| Administrative Class (Ref.) | - | - | - | - | - |
| Professional/Executive Class | 1.440 (0.23)* | 1.625 (0.29)** | 1.646 (0.29)** | 2.124 (1.12) | |
| Clerical/Support Class | 0.781 (0.29) | 0.974 (0.39) | 0.971 (0.39) | 1.222 (2.01) | |
| **Gender** | | | | | |
| Male (Ref.) | - | - | - | - | - |
| Female | 0.009 (0.01)*** | 0.009 (0.01)*** | 0.010 (0.01)*** | 0.010 (0.01)*** | 0.010 (0.01)*** |
| **Education** | | | | | |
| No post-secondary education (Ref.) | - | - | - | - | - |
| Post-secondary education | 0.663 (0.11)* | 0.677 (0.11)* | 0.674 (0.12)* | 0.684 (0.12)* | 0.680 (0.12)* |
| **Marital Status** | | | | | |
| Married (Ref.) | - | - | - | - | - |
| Not-married | 2.669 (0.42)*** | 2.705 (0.44)*** | 2.701 (0.44)*** | 2.707 (0.44)*** | 2.691 (0.44)*** |
| **Age** | 0.932 (0.01)*** | 0.934 (0.01)*** | 0.933 (0.01)*** | 0.934 (0.01)*** | 0.933 (0.01)*** |
| **Decreasing Subjective Status** | | 0.942 (0.05) | | 0.985 (0.10) | |
| **Subjective Status Groups** | | | | | |
| Higher status (Ref.) | | | - | | |
| Middle status | | | 0.834 (0.15) | | |
| Lower status | | | 0.766 (0.17) | | |
| **Material Class X Subjective Status** | | | | | |
| Administrative X Subj status (Ref.) | | | | - | |
| Professional/Executive X Subj Status | | | | 0.937 (0.11) | |
| Clerical/Support X Subj Status | | | | 0.947 (0.25) | |
| **Class-Status Combinations** | | | | | |
| Administrative-Higher status (Ref.) | | | | | - |
| Administrative-Middle status | | | | | 0.902 (0.23) |
| Administrative-Lower status | | | | | 0.890 (0.40) |
| Professional-Higher status | | | | | 1.892 (0.53)* |
| Professional-Middle status | | | | | 1.335 (0.31) |
| Professional-Lower status | | | | | 1.358 (0.32) |
| Clerical-Higher status | | | | | - |
| Clerical-Middle status | | | | | 2.038 (1.16) |
| Clerical-Lower status | | | | | 0.546 (0.27) |
| **Karlson, Holm, Breen-Mediation Analysis** | | | | | |
| **% of Material social class mediated by Subjective social status** | | | | | |
| Higher Class | | - | - | | |
| Middle Class | | -19.05% | -21.72% | | |
| Working Class | | 83.33% | 86.83% | | |

Source: Wave 5 of the Whitehall II Study ° p > 0.10 * p < 0.05, ** p < 0.01, *** p < 0.001. Entries represent the results of separate fixed effects logistic regression models
